# In and out among the Hospitals

**Published:** 1889-01-05

**Authors:** 


### In and Out Among the Hospitals.

By a Secretary of Ten Years' Standing.

Copies of Annual Reports and Rules, Accounts of Meetings, Changes in the Staff, &c., should be addressed The Editor, The Lodge, Porchester Square, W.

### THE SHEFFIELD GENERAL INFIRMARY.

The ninety-first report of this infirmary, which has just been published, does not go far into particulars of the work done during the past year; we, however, find that there were 1,812 patients treated in the wards, and 3,409 as out-patients. There was a daily average of 160 patients resident throughout the twelve months ending Midsummer last. The total of receipts was £8,652 4s. 10d., and of expenditure, £8,829 4s. 11d.: this gives the cost per bed occupied, after deducting 1s. for each out-patient and extraordinary expenditure, £53 9s. 7d. A considerable addition has been made to the medical staff by the appointment of two assistant physicians, two assistant surgeons, and another resident surgeon. The Overend Convalescent Fund has enabled 115 patients to be sent to convalescent homes. There are 44 cots devoted to children out of the 200 beds available in the infirmary.

### THE ADELAIDE HOSPITAL, DUBLIN.

The fundamental principle of this hospital is that only patients of the Protestant Reformed Church are admitted into its wards. The hospital was established thirty years ago on these restricted lines, and up to the present time this rule has been rigorously carried out, but it is gratifying to find that as regards the dispensaries, the committee have resolved to treat as out-patients persons of every religious denomination. A large amount of work was done last year, the daily average number of beds being 92, and altogether 1,170 patients were treated in the wards. This is the largest number recorded at this institution, and the public have shown their appreciation by liberally contributing to the funds. The income was £6,767, and the expenditure £6,088; so the committee were able to considerably reduce their little debt. Deducting 1s. per out-patient and extraordinary expenditure, the cost per bed occupied amounts to £67 5s. 5d. Certain wards are reserved for pay patients, who contributed £819 to the funds of the hospital. The nurses' home, opened last year, has proved of much value, and the services of the trained nurses sent out to private cases are much availed of.

### THE ROYAL HANTS COUNTY HOSPITAL.

During the last twelve months the number of patients under treatment was 1,545, of whom 621 were admitted as in-patients and 924 as out-patients. The receipts amounted to £5,111 9s. 9d., which is a slight decrease on last year, and the expenditure was £4,291 18s. 7d.; deducting 1s. per each out-patient, and the extraordinary expenditure, the cost per bed occupied was £58 15s. 4d. Some dimunition of income, attributable to the fact that the committee accepted the offer of the Chancellor of the Exchequer for the conversion of Consols will be felt in the future, and also the unavoidable loss caused by the reduction of Four per Cents. to Three and a-Half per Cents. The committee refer with much satisfaction to the development of the system of private nursing as undertaken by the hospital, and Mrs. Suckling comes in for high praise for the active part she has taken in the matter. Nurses are sent out at a guinea a-week, which cannot be considered a high rate. Most of the hospital buildings at Winchester are very old, and have few modern appliances, but the committee have lately established a very perfect system of fire apparatus, which has been fitted up under the supervision of the chief officer of the Metropolitan Fire Brigade. Mr. R. Moss, M.P., still maintains at his own expense an ambulance omnibus, which is not only a great convenience, but of much comfort to the patients. The hospital accounts are audited by two governors, but the committee have taken the wise course of placing all trust funds with the Official Trustee of Charitable Funds under the Charity Commissioners.

### LEEDS GENERAL INFIRMARY.

It is interesting to analyse the sources of income at this, one of the most important provincial hospitals. The total receipts for the year were £17,339, and this includes the munificent sum of £3,646 from the workpeople of Leeds. Ten years ago the sum raised from this source was a little over £1,000, but it has gradually increased, and last year exceeded the previous twelve months by £572. The ordinary annual subscription list is a large one, and amounts to £3,758. The only source of income that shows any serious falling-off is Hospital Sunday, and last year there was a decrease of £302. This is not what one would have expected in Leeds, which has had so many famous and philanthropic divines. The receipts also show a sum of £152 from paying patients. The total amount of ordinary expenditure was £16,187, being £704 in excess of the previous year, but this extra expenditure is small, considering the greatly increased number of patients treated. The daily average number of beds occupied was 275; deducting 1s. per each out-patient and extraordinary expenditure, the cost per bed occupied was £52 6s. 9d. The total of in-patients was 4,601, and out-patients 26,647; 220 patients were sent to convalescent homes during the year, of which number 157 went to the Convalescent Hospital at Cookbridge. A new convalescent hospital for patients from the Leeds Infirmary has been provided in the grounds of the Cookbridge Hospital. The new building, which has cost over £5,000 and contains 42 beds, has been presented by Mr. John North. This is therefore virtually an extension of the infirmary, making the total number of beds available 362 So great, however, has been the pressure on the present accommodation that the Board feels that before long it will be necessary to again build, in order to keep pace with the ever-increasing population of Leeds, an isolation building for infectious cases; another children's ward, and a new laundry are much required, and the Board only await an improvement in their financial position to commence the necessary work.

### SUNDERLAND AND BISHOPWEARMOUTH INFIRMARY.

This is one of the provincial hospitals in which a great interest is taken by the working community. The way in which collections are made, entertainments got up, and other means adopted for increasing the revenue of the charity is a recognition on the part of the working man of his appreciation of the institution. The zeal and liberality displayed proves that the charity enjoys the confidence of those who use it. Ten years ago the amount received from the workmen's representatives and committees was £1,388, and in the twelve months ending June last it was £2,842. The total receipts were £6,467 18s. 4d., and the expenditure £6,818 14s. 9d. Deducting 1s. per each out-patient, and the extraordinary expenditure, the cost per bed occupied was £39 11s. 9d., the daily average number of patients resident being 124. Three thousand nine hundred patients were treated in the twelve months, of whom 1,707 were received within the wards.

